# Reflections on qualitative research: Lessons learned to ensure research integrity

**DOI:** 10.4102/safp.v67i1.6082

**Published:** 2025-04-30

**Authors:** Deidré Pretorius, Khyati Dama, Michelle Erasmus

**Affiliations:** 1 Department of Family Medicine, School of Clinical Medicine, Faculty of Health, University of the Witwatersrand, Johannesburg, South Africa; 2 Unit of Undergraduate Medical Education, School of Clinical Medicine, Faculty of Health, University of the Witwatersrand, Johannesburg, South Africa

**Keywords:** qualitative research, research integrity, trustworthiness, credibility, supervision, Jasper’s reflection model, student-supervisor relationship

## Abstract

This article focusses on teaching medical students qualitative research in a practical setting of a study about sexual health. Insufficient training, research deviating from the proposal and context-related factors contributed to compromising trustworthiness of the study. The article provides a background to the study and reflects on factors that contributed to the breach of research integrity after the research supervisor questioned the credibility of findings. A semi-structured interview schedule was used, and data analysis exposed flaws. The team used Jasper’s reflective cycle to change a crisis into a learning opportunity. The action plan triggered round two of data collection, followed by another reflective cycle.

**Contribution:**

The supervision role and student knowledge and attitude contributed to the undergraduate research project. Until a research report is submitted or data published, researchers can learn from the process to produce reliable results and rigorous ethical research.

## Introduction

**FIGURE 1:**
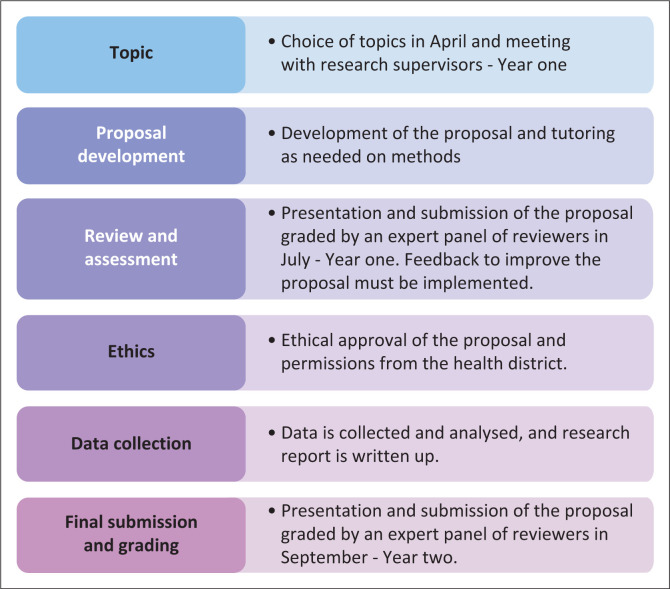
The undergraduate research process.

Undergraduate medical students are trained in research methods and ethics as part of the standard curriculum. As partial fulfilment for an undergraduate medical degree at the University of the Witwatersrand, South African students must work on a research project to apply the knowledge obtained from lectures (4 h) and self-directed learning from resources available to them. Students have two academic years to complete the research in their fourth and fifth year of training. Academics from all the disciplines in the Faculty of Health suggest topics and the students select topics aligning with their interest (Figure 1). The topics include sexual and reproductive health. The students have the liberty to formulate aim and objectives and choose the appropriate method, unless the project is part of a bigger study. The students have 2 years from choosing a topic to submitting a research report (Figure 1). The research supervisor must guide the students to develop an appropriate expert panel reviewed proposal.

Six undergraduate medical students designed research using a qualitative method. Although being undergraduate medical students, they were adult student-researchers aged between 23 years and 28 years old. All the students had previous bachelor or postgraduate degrees before they entered a graduate entry medical programme to start clinical training. This study set out to determine perspectives and understandings of the adult patients in primary care, regarding their sexual health, dysfunction and their associated help-seeking strategies.

There were at least three face-to-face sessions and online sessions discussing the qualitative method, pitfalls and process in the development of the proposal. The proposal was approved with a step-by-step data-collection process and analysis plan.

The qualitative study provided a platform to explore patients’ real-world experiences, behaviour patterns and attitudes.^1^ Upon reporting phase of the research results, it became clear that research integrity was not maintained. As reflection is considered an educational notion, it was the logical sequel to optimise professional practice and educational standards.^2,3^

There are various reflection models, and the authors chose the Jasper’s reflection model, namely the three stage Experience, Reflection and Action (ERA) cycle.^4,5,6^ The model is on-action reflection and focuses on the experience of what occurred and then reflect on thoughts and feelings associated with the experience, as well as next steps.^5,6^ Taking action is the final element in the cycle and focuses on what to do to get a different result in future.^5,6^ There is no limit on the reflective cycle, and it is recommended that after action was taken, a new reflective cycle is initiated.^5,6^

Using Jasper’s^5^ reflective model, this article reports on awareness of trust violations, questionable data collection and presentation and guidance on research and supervision practices. The reflective cycle will follow a short summary of the planned research to contextualise the disconnect between planning and the experience.

## Method

The study employed an explorative qualitative study design using a semi-structured interview schedule. The theoretical underpinning of this research was that of an inductive enquiry.^7^

The setting was the Lilian Ngoyi Community Clinic that serves approximately 90000 patients annually. Purposive sampling is common in qualitative research, but because of the sensitive nature of the research topic, with little to no screening for sexual dysfunction in primary care, the researchers relied on a convenience sample.^1^ Patients presenting for routine follow-up were informed about the study and asked if they would volunteer to participate. As the students do not work in these clinics yet, there is no conflict of interest or potential bias regarding the researcher–patient relationship. The inclusion criteria were clinically stable (meaning no medical emergencies, and patients within the standard thresholds of vital parameters, such as temperature, heart rate, respiratory rate, blood pressure, mental status and oxygenation) adult men and women consulting at Lilian Ngoyi Community Clinic who can speak English.

### Data collection

The semi-structured interview explored the patients’ background such as ethnicity and cultural beliefs, gender and norms that could influence their perceptions on sexuality and sexual functioning.^8,9^ It also explored their understanding of sexual dysfunction, as well as where or how they will seek help. It further included a demographic questionnaire covering patient characteristics. The interview schedule was based on previous research findings.^8,9,10,11,12^ The first few questions covered the setting in which the person was raised, cultural and religious value set to understand the context of the participant.^13^ These questions were followed by more sensitive questions such as were sexuality or sexual problems ever discussed at home; what they understand as being a ‘sexual problem’ (probes covered infectious diseases, family planning and reproductive health, familiarity with sexual functioning problems, low sexual desire symptoms, orgasm challenges, etc., sexual orientation and where would people get help for it); awareness of cultural practices or remedies for sexual problems and lastly, where would a person go if the person had a sexual problem and reason for the choice.

#### Planned data-collection process

The expectation was that data collection will be done as planned. The research proposal had a clear description of the data-collection process. As training occurred a few months prior to data collection, another training session or refresher was scheduled for students on qualitative data collection. The approved proposal had a pilot study planned. The students had to self-select roles as interviewer, scribe and capturing field notes based on personal communication strengths. Participants were invited to listen to their recordings after the interviews and rectify any responses if necessary. The students were given a guide for transcription of their interviews.^14^ Thematic analysis^15^ was done manually by the students using agreed upon colour highlighters to identify codes and categories. These were then again grouped, and themes emerged. The students claimed they reached data saturation as no new data were obtained. When the research supervisor reviewed the submitted transcripts using Max QDA 2020, the supervisor realised that there were various discrepancies. Upon engagement with the students about these discrepancies, the supervisor realised there was a disconnect between verbal and written events. This triggered the reflection cycle.

#### Jasper’s reflective model: Experience

A colleague stepped in when the primary supervisor was hospitalised. The student-researchers claimed to be too busy with clinical work and agreed to 60-min online refresher training. The students considered their first two interviews as a pilot study. They never critically evaluated the interviews. By the time the primary supervisor returned, the student-researchers already completed the data collection. Students did not engage with recorded data at any point before analysis.

**FIGURE 2:**
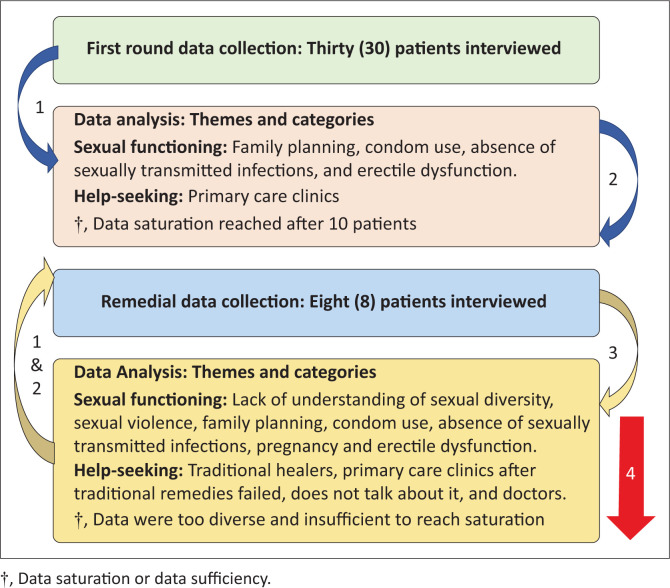
The reflection cycle during the research process and emerging themes.

Claiming time pressures, the researchers deviated from initial plans and interviews occurred without the agreed upon roles. Each member interviewed several participants. No diary or fieldnotes were kept. Patients had the opportunity to listen to the recordings at the end of the interviews. When the research supervisor requested transcriptions, only a few ‘transcriptions’ could be provided, and it was paraphrased text. Transcription errors undermine the trustworthiness of research findings.^16^ Comparing recordings with transcripts, the number on the reported interviews, transcripts and content of transcripts and recordings did not match. To fast-track the data-collection process, the students adjusted and used the interview schedule as a researcher administered questionnaire. The interview schedule changes made the questions very personal and without context. They asked multiple closed-ended questions and leading questions favouring answers supporting Eurocentric help-seeking strategies. To understand these methodological violations (Figure 2), the research team used a structured reflective approach: (1) Experience; (2) Reflection; (3) Action; (4) Next phase of reflection cycle.

#### Jasper’s reflective model: Reflection

Both trustworthiness and research integrity were compromised questioning the research values or the students.^17^ Being undergraduate students, the integration between knowledge, understanding and application could contribute to lower ethical decision-making skills or ethical competency.^17^ Unfortunately, learning activities were theoretically developed, but not enacted and re-enacted,^18^ as the students failed to do the planned training, pilot study or simultaneous data collection and analysis.

When the trustworthiness and credibility^19^ of the research were questioned, emotional responses varied between shock and astonishment, indignation, doubt and anger. Initially, the students did not believe that research integrity was breached. Feelings varied between supervisor self-blame, loss of mutual trust (important in the teacher–student or supervision relationship), embarrassment and fear. Some students were defiant, and others tried to placate all. Some students felt they did nothing wrong, while others realised they did not meet the requirements for the task that caused the process to derail. Although nobody blamed the other openly, the undercurrent of blame was palpable. Of note, there were students in the group who recognised the shortfalls and were humbled by their first full research experience. It was flawed research, but the students were overwhelmed by other academic requirements, and the research became a minor competing requirement. In retrospect, we initially repressed our feelings in the interest of academic progress (the institutional objective). Feelings were verbalised after meeting the institutional objective of the submission of a research report. This also formed part of the reflection process.

#### Possible external contributing factors

The unforeseen absence of the research supervisor in the initial phase of research contributed to the challenges experienced. The research participants who volunteered for interviews were male and female patients between 23 years and 54 years. They originated from townships such as Diepsloot, Khayelitsha and rural areas in KwaZulu-Natal (now residing in Soweto). The patients identified with the Christian faith and came from diverse cultural backgrounds, namely, Zulu, Sotho, Xhosa and Venda. The expectation was that medical students would be able to navigate a conversation with a patient as communication skills are part of the core curriculum from their first year of study. Nevertheless, we were aware that it may be more difficult with older patients, but the assumption was that a person would not volunteer after being informed on the nature of the study, if the person was not comfortable talking to a student. If the participant was comfortable to talk to the student-researchers knowing what the focus of the study is, and the interview guide was used as it was developed, there was little opportunity for patient–researcher interaction to go wrong. However, the effect of the COVID-19 pandemic on student–patient engagement must be considered. The group of students conducting this research completed a large part of their medical degree online. This might have affected their ability to converse with patients, as they had less exposure to patient engagement and communication despite tutorials on communication skills. Their first exposure to the clinical world was in 2022 when they commenced with the research. In planning the research and methods, there was no way any person could anticipate what the impact of COVID-19 could be on training.

The study setting also played a role in events. This area is a low socio-economic urban area and serves individuals in and around the larger area of Soweto. Data collection was postponed a few times because of periodic civil and labour unrest around the clinics.^20,21^ Safety of students is paramount. Unfortunately, these delays added to stress meeting timelines for submission of the research report.

#### Jasper’s reflective model: Action

Based on reflection, action plans included a new round of data collection. Students had to meet the submission timelines for their reports and thus it was not possible to do full data-collection cycle but repeat the process the way they were supposed to. The second round improved the process and data greatly, and transcriptions and interviews aligned (see Table 1 for the data quality on first and second round).

**TABLE 1:** Examples of excerpts and learning gained.

Round one interviews (Various interviewers)	Round two interviews (Two dedicated interviewers)
**Sexual health and/or challenges:** R4: ‘What do you understand as a sexual problem?’ LN5: ‘When you don’t use condoms and fall pregnant’. R4: ‘It is common?’ LN5: ‘It is important to see a doctor because some are sexually active, and they can get more information’. R4: proceeds with a new question. R6: ‘What do you understand as a sexual problem? Are you aware of problems with contraception? Any other diseases? Have you ever used contraception before? Did you plan to have children? How common do you think sexual problems are? Do you believe seeing a doctor about sex and sexual health is important?’ LN9: ‘… [*Quiet*] …Those diseases that you get when sleeping with women. I am worried that it has happened with me here because it is sore there (points down to penis), but I don’t know what it is’. R6: ‘Do you know about any diseases?’ LN9: ‘The HIV’ R6 proceeds with a new question.	**Sexual health and/or challenges:** R2^2^: ‘… And uhm, when I say the words “sexual problems” what do you understand about that?’ LN2^2^: ‘I understand maybe… if maybe if you’ve got sores in your private parts, maybe your husband or your partner maybe doesn’t get the right erections. Or maybe he’s got an early ejaculations. Or maybe STIs. Or maybe … something like that’. R2^2^: ‘Uhm so we’ve been talking about the word sexual problems, what do you understand when I say sexual problems?’ LN7^2^: ‘Sexual problems, hmmm what I understand is uhm uh uh if maybe you are forced by your partner that is a problem. If you are forced by a partner to have sex. When you don’t, you don’t want to or maybe when you raped outside there when you walking at night come across of the certain gang members and then yes and then another six [*sex*] is between the sex’s female and men, you know, there were people who what can I put it? Say it’s a woman, but his behaviour is behave [*behaving*] like a man there is the name of that where a men behave, we used to have a guy who was who was he was playing with ladies. So, that was a problem. The sex problem because he was a man and we knew him as a man, but he was playing with us’ R2^2^: ‘… And did you guys view that as a sexual problem?’ LN7^2^: ‘… what uh uh a male tend to be … Uhhhhh it is a problem I can say. So yeah, it is a problem. Because you see now that is allowed to be whatever you want to be those uh what they call them uh the bisex. All that yeah. So, it is because now we see many … eh … stories about the … the people who are bisex or that kind of they were harassed of the way they are. And then they end up being killed some way. So, I think that is a problem and even in the family, they need to accept that their child is like that and then it takes time. Others they don’t accept that especially the cultural people like Zulus they if they say there’s nothing like this in our family, you are a curse in our family’.
**Cultural practices:** R2: ‘You said earlier that your ethnic community, cultural group, or tribe was Sotho…. are you aware of some cultural or religious practices or remedies for sexual problems?’ LN5: ‘No, nothing’ R2 proceeds with a new question. R2: You said earlier that you are part of the Zulu community, are you aware of some cultural or religious practices or remedies for sexual problems? Tell me about it. LN11: ‘I think there is traditional medicine, but I don’t practice it. I come to the doctor’. R2 proceeds with a new question. R6: ‘You are part of the Xhosa community, are you aware of some cultural or religious practices or remedies for sexual problems? Tell me about it’. LN 13: ‘No no no. Nothing in my family’ R6: proceeds with a new question.	**Cultural practices:** R2^2^: ‘Uhm … Zulu, Sotho? Are you familiar with any cultural practices or traditional medicines for these problems?’ LN2: ‘Although I don’t use them, but I know there are. Yes, I know’. R2^2^: ‘Which ones do you know about?’ LN2^2^: ‘The mbiza maybe for men. And then, some, some, some they say it helps. And then maybe, I’ve got a problem with my womb, I can find a mbiza to clean myself; to cleanse myself. And I feel okay’. R1^2^: ‘Okay um … okay you said earlier that you are Zulu so in the Zulu culture do you have any like traditions or like things that you do for sexual health?’ LN5^2^: ‘No because there is no circumcision’. R1^2^: ‘Oh Okay’ LN5^2^: ‘Yes’ R1^2^: ‘Oh so you don’t have like initiation school’ LN5^2^: ‘No there isn’t. Besides girls, for girls only. But for males they don’t circumcise. It’s rare. Most of Zulu guys they circumcise at the clinics. But then their tradition they don’t circumcise’. R1^2^: ‘Okay. So, what do they do for the girls’ LN5^2^: ‘there’s these things that they do. I don’t know it. [laughs] I’ve never went there before. So, I do not know it’. R1^2^: ‘… and then do you have like any like remedies or treatments that people use for like sexual problems that they have’. LN5^2^: ‘Whoa! There is a lot but I do not know their names. [*laughs*]’.
**Sex education at home:** R7: ‘Do you discuss sexual problems at home?’ LN8: ‘No’ R7: ‘Thank you’	**Sex education at home:** R1^2^: ‘… when I talk about, uhm, sexuality and sexual problems was that ever discussed in your home?’ LN3^2^: ‘No … uh … no [*laughs loudly*]’. R1^2^: ‘You seem shocked, why?’ LN3^2^: ‘No no, we were just told like, don’t play with boys, just that, don’t play with boys. Interviewer: [*laughs*] … Don’t play with boys, like the other consequences, maybe you will find this and this, and maybe some … like I understand that maybe you can tell your child, that no, if you go with this route there are these consequences. If you try to do this, there are, there’s something that will happen. But try to do this, maybe it will help you in the fu… in the near future. But then we didn’t have that kind of discussion. It just told, don’t play with boys’. R2^2^: ‘OK. And when you say consequences, what do you, what are you referring to?’ LN3^2^: ‘Yoh. There are so many. Pregnancy, number one. You get pregnant at the early age. All your things just go poof. All your dreams just go poof. Yeah. You just don’t realise that some of the things, that maybe when you realise, no, if maybe I had, I tried to maybe to control myself and my feelings, maybe I would’ve done this and that and that. Yeah’.
**Help seeking:** R 1: ‘Do think that going for these sexual problems, to a doctor, is important?’ LN1: ‘Yes, I’ll go definitely go to a doctor’. R1: ‘Doctor. Where? Where would you go?’ LN1: ‘Maybe a clinic? Or maybe a private doctor? But as long as, as I’m going to be comfortable in the place to talk about my my my …’ R4: ‘Do you believe that seeing a doctor about these problems is important?’ LN5: ‘Yes’ R4: ‘And again, hypothetically, if you had one of these problems, would you seek help? Would you go to a doctor?’ LN5: ‘Yes, definitely’.	**Help seeking:** R1^2^: ‘So, if you have sexual problems, where would you go?’ LN6^2^: ‘I will start at sangoma [*traditional healer*] first’. R1^2^: ‘Why would you go there first?’ LN6^2^: ‘That’s where I believe in’. R1^2^: ‘And would you just go to the sangoma? Would you go anywhere else?’ LN6^2^: ‘If the Sangoma is not perfect, then I can come here to Men’s Clinic actually’ R1^2^: ‘Why would you come to the men’s clinic?’ LN6^2^: ‘Because that’s where I will feel comfortable, because I’ll be talking to another man’.

R, researcher followed by researcher number; LN, Lilian Ngoy followed by patient number; STI, sexually transmitted infection; HIV, human immunodeficiency virus.

Second round interviewers and patients are indicated with superscript 2(^2^).

## Reflection cycle repeated

The immediate reaction was a form of catharsis. All the student-researchers were relieved that they could submit their research report to meet the academic requirement. One student mentioned that they redeemed themselves. Unfortunately, only the authors were willing to participate in a formal second round of reflection. The experience of the data collection on round two was positive, and it was clear that learning took place. This also resulted in feelings of excitement about the learning and findings. As there was no data saturation or data sufficiency reached in round two, one aspect piqued our interest from the second round of interviews: patients did not only misunderstand sexual dysfunction, but linked it with sexual violence, human immunodeficiency viruses (HIV), rape and sexual orientation. Although the research cannot conclude on this, future research can explore it.

Reflection would not be complete without considering all factors that could contribute to the events. A research supervisor takes ultimate responsibility – ‘a good supervisor is one who knows how to productively manage the inevitable tensions that arise between people, the research itself and the institutional objectives’.^22,23^ The students were already delayed by the civil unrest and the initial absence of the supervisor, and they failed to use the support that was arranged.^24^ Not knowing the students changed the arrangements, the supervisor acted in good faith that the students followed the required planning and they understood the research expectations, and had the necessary capability to apply what they learned.^25^ The supervision role was that of a group supervisor based on a relationship with the students, cultivating relationships with each other which were supposed to serve as peer support and oversight.^26^ Undergraduate students may not have the confidence to use the inter-student relationships to give the oversight or contribute to scholarly competence, but emotional support can complement the supervisory task. In this research, the presumed peer support was more peer pressure to complete an academic exercise and thus a misconception from a supervisor’s point of view. One forgets that undergraduate students may not feel comfortable or even be fearful to express their needs.^27^

De Beer and Mason^28^ mentioned how the supervisory role expanded in postgraduate supervision to include the role of an advisor, a quality controller and that of a guide or mentor. This is even more true for supervising research for undergraduate students. Quality control stops questionable results reaching the public domain and serves as a rich platform for reflection and learning.

## Conclusion and future action

Qualitative research can be challenging for students. Students at the entry level in research require meticulous supervision and fact checking. Research often presents unexpected challenges and can be avoided with preparation and personal accountability. The student–supervisor relationship is paramount in the research process. Teaching research integrity can help young researchers to navigate the research world. It may trigger them to be more confident to express concerns when they experience challenges. They may also be more confident to express the lack of competencies and ask for guidance when they are unsure. Communication in group research can improve peer support, peer oversight and contribute to research integrity.

The revised medical curriculum starting in 2025 dedicates more time to research and research ethics. More importantly, the research supervisor must not hesitate to stop a research project and encourage introspection. Until a research report is submitted or data published, researchers can learn from the process to produce good results and rigorous ethical research.
